# Decreased mean platelet volume predicts poor prognosis in metastatic colorectal cancer patients treated with first-line chemotherapy: results from mCRC biomarker study

**DOI:** 10.1186/s12885-018-5252-2

**Published:** 2019-01-07

**Authors:** Jinjia Chang, Guangyi Lin, Min Ye, Duo Tong, Jing Zhao, Dan Zhu, Qihe Yu, Wen Zhang, Wenhua Li

**Affiliations:** 1 Department of Medical Oncology, Shanghai Cancer Center, Fudan University, Shanghai, People’s Republic of China; 2 Department of Oncology, Shanghai Medical College, Fudan University, Shanghai, 200032 China; 3 Shanghai medical college, Fudan University, Shanghai, 200032 China; 4 Department of Pathology, Shanghai Cancer Center, Fudan University, Shanghai, 200032 China

**Keywords:** MPV, Metastatic colorectal cancer, Chemotherapy, Biomarker

## Abstract

**Background:**

Metastatic colorectal cancer (mCRC) is a major cause of death of malignant tumor and the valuable prognostic biomarker for chemotherapy is crucial in decreasing mortality. Previous studies have proved the prognostic value of the mean platelet volume (MPV) in survival of primary operable CRC patients. However, the prognostic impact of MPV in mCRC is still unclear. In this study, we aimed to clarify the prognostic role of MPV in mCRC undergoing standard first-line chemotherapy.

**Methods:**

From January 2012 to December 2016, we conducted a retrospective clinical study included 264 mCRC patients (NCT03532711). All the enrolled patients received the standard oxaliplatin-based or irinotecan-based chemotherapy. The association between the baseline MPV and clinicopathological features were examined.

**Results:**

Univariate analysis revealed that decreased MPV, the platelet counts (PLT), platelet-to-lymphocyte ratio (PLR) and the platelet crit (PCT) were significantly associated with inferior overall survival (OS) (*p* < 0.05). On multivariate analysis, elevated PLR was significant prognostic factors for OS, with hazard ratios of (HR:1.006, 95% CI:1.001–1.011, *p* = 0.01) while MPV was not, respectively (p < 0.05).

**Conclusions:**

Our study demonstrated that the baseline MPV level may act as a predictive factor for survival in mCRC patients undergoing standard chemotherapy.

**Trial registration:**

This study was retrospectively registered in date May the 20th 2018. The registration number (TRN) of this study was NCT03532711.

**Electronic supplementary material:**

The online version of this article (10.1186/s12885-018-5252-2) contains supplementary material, which is available to authorized users.

## Background

Colorectal cancer (CRC) is the third most common malignancies in males and second most common in females worldwide. The fatality rate ranks the fourth and the third for males and females, respectively [1]. Standard chemotherapy remains the most effective therapy for patients with metastatic colorectal cancer (mCRC) [2]. However, some patients do not benefit from chemotherapy but are exposed to the adverse effects [3]. Therefore, there is an urgency to develop reliable prognostic biomarkers for mCRC patients receiving standard chemotherapy.

In our previous study, we set up a clinical trial to evaluate potential biomarker of mCRC patients undergoing routine chemotherapy (mCRC biomarker study, https://www.clinicaltrials.gov, NCT03532711). One of our published paper indicated that patients carrying several SNP combinations may benefit more from the first-line chemotherapy such as FOLFOX/XELOX regimen. Our results suggested that the combination of SNPs may predict the therapeutic efficacy of the first-line chemotherapy for mCRC patients [4]. In the current study, we aimed to identify prognostic mCRC biomarkers that are low-cost and easily obtainable via routine blood counts.

It is well known that platelet activation acted as an active role in cancer progression and metastasis [5]. MPV which measures the average size of platelets in the blood might act as a marker of platelet activation [6–8]. MPV and PLT count are two main characteristics to evaluate platelet activation. Previous studies have revealed that MPV or its related factors such as PLT or PDW were crucial in many malignancies progression [9–12]. Notably, most of the research focused on early stage tumor or primarily operable cancer, few of them discussed the value of MPV in predicting late stage cancer patients’ outcome [12, 13]. For example, the predictive value of PLT combined with MPV for overall survival in mCRC undergoing chemotherapy has not been yet investigated.

Herein, by conducting a clinical trial (NCT03532711), we aimed to investigate the effect of MPV levels on pathological factors as well clinical outcome of mCRC patients undergoing routine chemotherapy.

## Methods

### Study description

A retrospective observational clinical study was conducted to find the biomarkers in chemotherapy regimens for first-line chemotherapy for mCRC (NCT03532711). Patients with histopathologically confirmed mCRC who had at least one measurable lesion were enrolled. Two hundred sixty-four mCRC patients were enrolled in this clinical study and assigned to receive standard first-line chemotherapy such as FOLFOX/XELOX/FOLFIRI regimen according to the investigators’ suggestion. The primary end point was objective response rate. Secondary end points were overall survival and progression-free survival. All patients collected pre-treatment complete blood cell counts. Treatment efficacy was regularly evaluated, and the best response efficacy was recorded according to the RECIST 1.1. This retrospective study was approved by the Ethical Committees of Fudan University Shanghai Cancer Center (Ethics Number: 1203108–10).

### Biochemical measurement

We collected the peripheral venous blood sampling from each patient at the baseline of therapy. Whole blood samples were collected in EDTA-containing tubes for validation of platelet counts, white blood cell (WBC) and hemoglobin.

### Statistical analysis

We used the Receiver-operating characteristics (ROC) curve to select the optimum cut-of value of MPV. The Student’s t-test was used to determine the significance of between-group differences were determined. The Kaplan–Meier method was used to describe analysis of survival curves. Independent factors were identified by multivariate Cox proportional hazards modeling. A value of *p* < 0.05 was considered of significance. All statistical analyses were conducted using SPSS Statistics version 22.0.

## Results

A total of 264 metastatic mCRC patients were enrolled in this clinical study (NCT03532711). One hundred seven patients (40.5%) were women and 157 patients (59.5%) were men, and the median age was 55.5 years old. 165 (62.5%) patients suffered from colon cancer while 99 (37.5%) patients were with rectal cancer. For tumor metastatic pattern, 146 of 264 patients (55.3%) were with synchronous metastasis and the rest 118 patients were with metachronous metastasis.

**Fig. 1:**
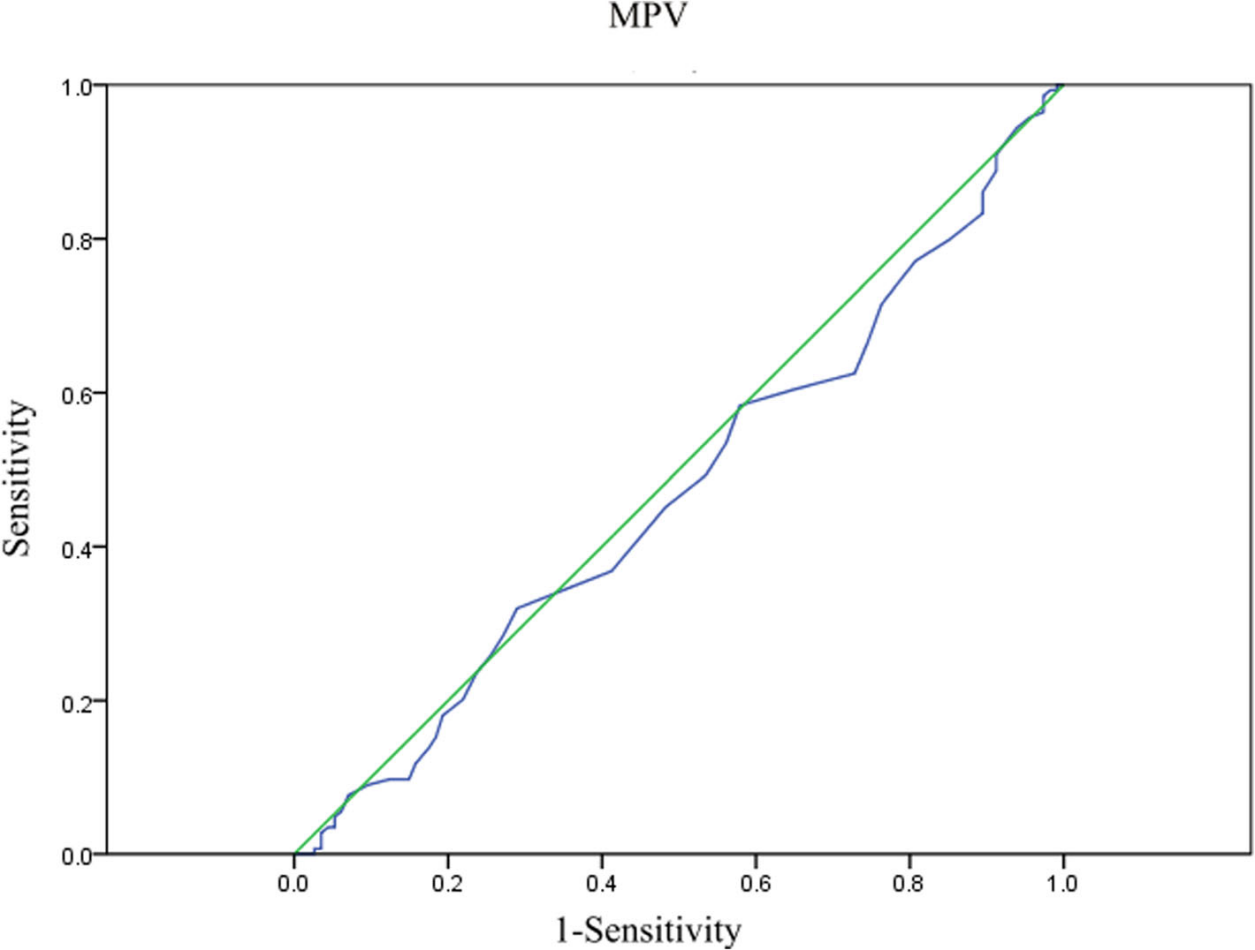
Optimized cut-off was determined for MPV using standard ROC curve analysis

MPV level were recorded in all the patients at baseline. As shown in Additional file 1: Figure S1, 108 patients were out of normal range while 156 patients were in the normal range. We used a ROC curve to determine the optimal cut-off value of MPV. As a result, an MPV of 9.75 fL yielded maximum combined sensitivity and specificity (see Fig.1). It is showed that MPV predicted cancer prognosis with a sensitivity of 0.625 and a specificity of 0.272 (AUC = 0.473, 95% CI: 0.402–0.544, *p* = 0.459). Therefore, we could clearly classify the patients into two independent groups: patients with MPV ≥ 9.75 fL and patients with MPV<9.75 fL. In this study, 173 (65.6%) patients were with MPV ≥ 9.75 fL and 91(34.4%) patients were with MPV<9.75 fL.

Table 1 and Table 2 showed the relationship between MPV and clinical characteristics. Some of the clinicolaboratory characteristics (PLT, PCT, PLR, hemoglobin, ANC, WBC, PLR, MPV/P, PDW/P and Efficacy) were demonstrated closely correlated with MPV. The other factors including age, gender, location, regimen, histology and metastasis pattern showed no difference between the two groups.

**Table 1:** Baseline characteristics of patients with mCRC according to MPV levels

Variables	Total n (%)	MPV ≥ 9.75	MPV<9.75	*P* value
Age (years)				0.6329
≤65	209(79.2%)	135(78.0%)	74(81.3%)	
>65	55(20.8%)	38(22.0%)	17(18.7%)	
Gender				0.9753
Male	157(59.4%)	103(59.5%)	54(59.3%)	
Female	107(40.6%)	70(40.5%)	37(40.7%)	
Location				0.5950
Colon	164 (62.3%)	106(61.3%)	59 (64.8%)	
Rectum	99 (37.7%)	67 (38.7%)	32(35.2%)	
Regimen				0.3024
Oxaliplatin	125(46.2%)	86(49.7%)	39(42.9%)	
Irinotecan	142(53.8%)	87(50.3%)	52(57.1%)	
Histology				0.3593
adenocarcinoma	239(90.5%)	161(93.1%)	78(85.7%)	
mucinous adenocarcinoma	15(5.7%)	7(4.0%)	8(8.8%)	
signet-ring cell carcinoma	4(1.5%)	2(1.2%)	2(2.2%)	
NK	6(2.3%)	3(1.7%)	3(3.3%)	
Metastasis pattern				0.4852
simultaneous	146(55.3%)	93 (53.8%)	53(58.2%)	
metachronous	118(44.7%)	80(46.2%)	38(41.7%)	
Efficacy				0.0166
CR + PR	84(31.8%)	65(37.6%)	19(20.9%)	
SD	132(50.0%)	81(46.8%)	51(56.0%)	
PD	27(10.2%)	18(10.4%)	9(9.9%)	
NK	21(8.0%)	9(5.2%)	12(13.2%)	

Abbreviations: *NK* not known, *CR* complete response, *SD* stable disease, *PD* progression disease

**Table 2:** Association of MPV with the clinicolaboratory characteristics of patients with mCRC

Variables	MPV ≥ 9.75	MPV<9.75	P value
PLT (× 10^9^/L)	226.82	287.81	0.000
PCT	0.25	0.27	0.079
PDW(%)	13.11	10.21	0.000
Hemoglobin (≤120 × 10^9^/L vs.>120 × 10^9^/L)	18.23 ± 1.49	25.33 ± 2.637	0.006
ANC (×10^9^/L)	4.53	5.25	0.016
WBC (×10^9^/L)	6.67	7.46	0.017
PLR	173.48	209.46	0.006
NLR	3.579	3.89	0.353
MPV/P	0.06	0.03	0.000
PDW/P	0.07	0.04	0.000

Abbreviations: *PLT* platelet count, *PCT* platelet crit, *PDW* platelet distribution width, *WBC* white blood cell, *ANC* neutrophil cell, *MPV* mean platelet volume, *NLR* neutrophil-to-lymphocyte ratio, *PLR* platelet-to-lymphocyte ratio, *MPV/P* MPV-to-platelet ratio, *PDW/P* PDW-to-platelet ratio

**Fig. 2:**
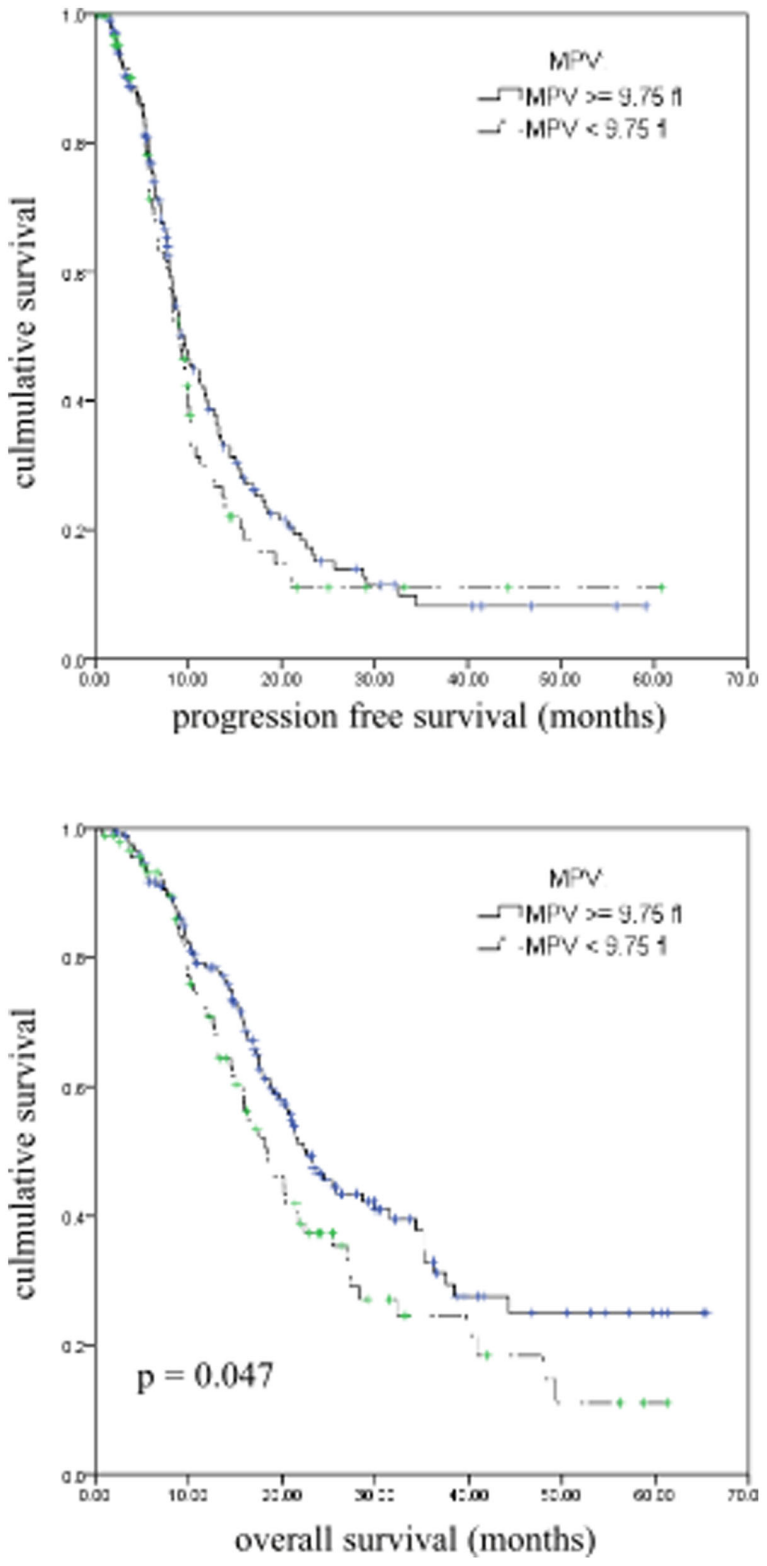
Influence of MPV levels on overall survival and progression free survival by Kaplan-Meier analyses. The Kaplan-Meier survival curve with log-rank analysis of OS showed statistical significance between curves of patients with MPV low level and high level in mCRC biomarker study (log-rank test)

The Kaplan–Meier method was used to describe analysis of survival curves. As is shown in Fig. 2, patients with low MPV levels had worse OS than those with high MPV levels (22.57 ± 1.813 months vs. 18.37 ± 2.132 months, *p* = 0.047). Our results demonstrated that mCRC patients with decreased MPV level have an inferior outcome than those patients with high MPV level.

Analysis of variance was performed to clarify the relationship between MPV and ORR. The results showed that ORR was 39.6% in the patients with high MPV levels and 24.1% in the patients with low MPV levels. In the univariate analyses, MPV and several clinicolaboratory characteristics (gender, hemoglobin, PLT, WBC, ANC, PCT, PLR, NLR and efficacy) were associated with PFS or OS ( see Table 3 and Additional file 2: Table S1). Those characteristics with a *p*-value less than 0.1 were included in the multivariate analysis. As a result, WBC, ANC, PLR, PLT and efficacy were independent factors for OS (see Table 3 and Table 4).

**Table 3:** Results of univariate analysis of overall survival in patients with mCRC

Variables	Hazard ratio	95%CI	P-value
Age (years) (≥ 65 versus < 65)	0.918	0.56–1.261	0.400
Gender	1.432	1.037–1.977	0.029
MPV (≥ 9.75 fL versus < 9.75 fL)	1.398	1.005–1.946	0.047
WBC	1.082	1.018–1.150	0.011
ANC	1.084	1.016–1.157	0.015
Lymphocyte	0.861	0.64–1.157	0.320
Hemoglobin (≤120 × 10^9^/L vs.>120 × 10^9^/L)	0.630	0.453–0.87	0.006
PLT	1.002	1–1.003	0.018
PCT	4.234	1.17–15.322	0.028
PDW	0.961	0.901–1.026	0.234
PLR	1.003	1–1.004	0.002
NLR	1.066	1.007–1.128	0.028
Efficacy	1.529	1.285–1.820	0.000

Abbreviation: see Table 1 and Table 2

**Table 4:** Results of multivariate analysis of overall survival in patients with mCRC

Variables	HR	95%CI	*P*-value
Gender	1.307	0.932–1.833	0.121
MPV (≥ 9.75 fL versus < 9.75 fL)	1.117	0.752–1.658	0.584
WBC	1.589	1.193–2.116	0.002
ANC	0.679	0.470–0.980	0.038
Hemoglobin (≤120 × 10^9^/L vs.>120 × 10^9^/L)	0.809	0.548–1.193	0.285
PLT	0.995	0.991–1.000	0.038
PCT	2.470	0.237–25.702	0.449
NLR	0.921	0.749–1.132	0.436
PLR	1.007	1.003–1.012	0.002
Efficacy	1.582	1.311–1.909	0.000

All variables with a *p*-value lower than 0.1 in univariate analysis were included in a multivariate statistical analysis

## Discussion

Altered MPV might be valuable prognostic biomarker for malignant tumor patient, however, MPV level and its relationship with the patient outcome remains unclear. For example, previous studies suggested that elevated MPV levels were proved significantly associated with better outcome in esophageal cancer [3], breast cancer [13] while other research indicated that reduced MPV were associated with worse outcome in lung cancer [14], renal cancer [15] and bladder cancer [16]. As for colorectal cancer, the patients with elevated MPV level, compared with those with normal level, were found had worse overall survival [17]. Notably, to our best of knowledge, there lacks the analysis of the survival impact of MPV in patients with mCRC treated with first-line chemotherapy. In this study, we aimed to analysis the correlation between pretreatment MPV level and overall survival in patients with metastatic CRC undergoing the first line chemotherapy regimens.

In order to study the issue by a more precise way, we conducted a prospective clinical study to characterize and evaluate biomarkers of chemotherapy in patients with mCRC in the first-line setting. Two hundred sixty-four patients were enrolled and they were treated with first line chemotherapy including FOLFOX/XELOX/FOLFIRI. Our results showed that the group of patients with decreased MPV level had worse overall survival than the group with high MPV level (shown in Fig. 2). In addition, we performed univariate analysis and found that MPV, together with platelet count, PCT and PLR, were associated with OS. Unlikely, PLR but not MPV was demonstrated as an independent prognostic factor for mCRC undergoing chemotherapy. In contrast with our study, Yu et al reported that operative CRC patients with elevated MPV level had poor overall survival compared with those with normal level. Our results indicated that the situation may be different in late-stage CRC compared with operable CRC [17].

The mechanisms underlying the association between MPV and mCRC patient outcome in is not entirely clear. MPV is an initial symbol of activated platelets [18]. Decreased MPV could be recognized as elevated expression of large platelets in inflammatory states [19]. Large platelets could release a variety of pro-inflammatory cytokines such as interleukin-6 (IL-6), which may improve tumor progression and metastasis [20]. It is well known that there is a confirm linkage between CRC and chronic inflammatory [21]. Several pre-treatment inflammatory indexes such as PLR and NLR were probed as predictors of prognosis and treatment efficacy in patients with metastatic colorectal cancer mCRC [22]. This may explain the reason that decreased MPV may result in mCRC tumor progression and shorter overall survival.

Our research has several limitations. First, our clinical study was a single-center retrospective study and more ethnic groups are warranted to confirm our results. Second, the mechanism of MPV in mCRC treated with chemotherapy should be further illustrated. For example, further investigation could be performed to clarify the association of decreased MPV and chemotherapy regimens such as oxaliplatin or fluorouracil.

## Conclusions

In conclusion, MPV and its related factor PLR were proved in the current study to act as a prognostic biomarker for mCRC patients undergoing first-line chemotherapy. Future studies need to be developed to study the underlying mechanism of MPV in mCRC progression.

## Additional files

Additional file 1: **Figure S1.** MPV level distribution of 264 patients. (PNG 78 kb)

Additional file 2: **Table S1.** Results of univariate analysis of progression free survival in patients with mCRC. (DOCX 16 kb)

## Abbreviations

HRs: Hazard ratios
mCRC: Metastatic colorectal cancer
MPV: Mean platelet volume
OS: Overall survival
PCT: Platelet crit
PLR: Platelet-to-lymphocyte ratio
PLT: Platelet counts
ROC: Receiver-operating characteristics
WBC: White blood cell
