# Supplementary figures and images for: Decreased mean platelet volume predicts poor prognosis in metastatic colorectal cancer patients treated with first-line chemotherapy: results from mCRC biomarker study
Source: BMC Cancer. 2019 Jan 7;19:15. doi: 10.1186/s12885-018-5252-2 (PMC6322328; doi:10.1186/s12885-018-5252-2)

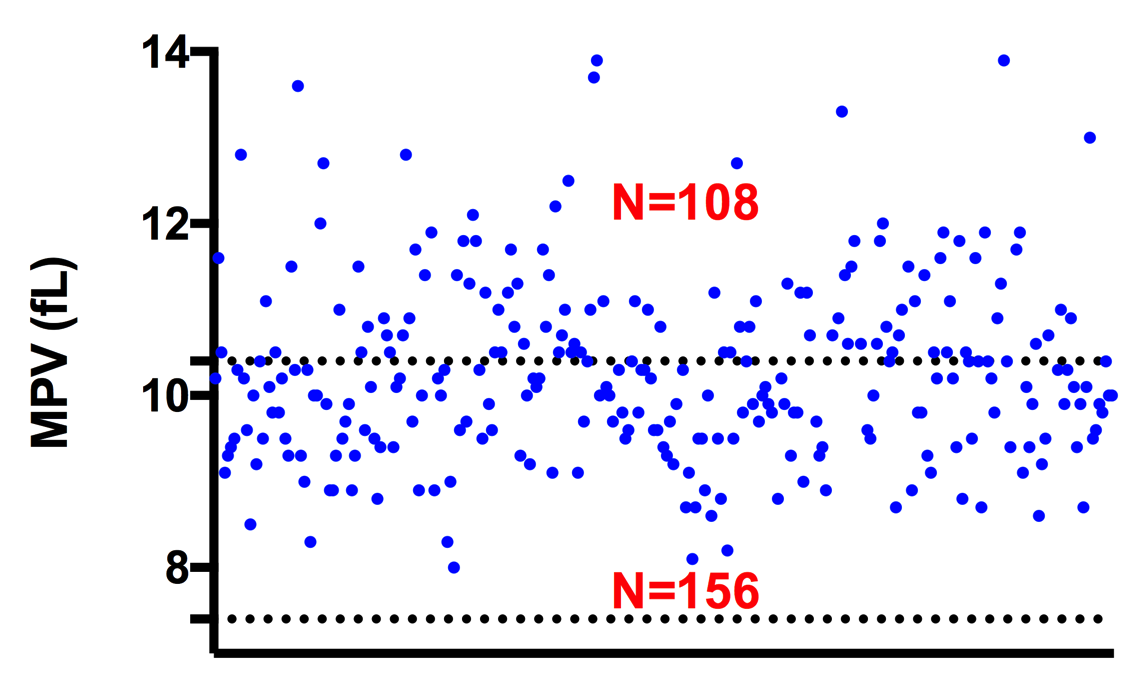

Supplement: Supplementary file 1 — Figure S1. MPV level distribution of 264 patients. (PNG 78 kb) [file 12885_2018_5252_MOESM1_ESM.png]
