# Supplementary material for: Decreased mean platelet volume predicts poor prognosis in metastatic colorectal cancer patients treated with first-line chemotherapy: results from mCRC biomarker study
Source: BMC Cancer. 2019 Jan 7;19:15. doi: 10.1186/s12885-018-5252-2 (PMC6322328; doi:10.1186/s12885-018-5252-2)
Supplement: Supplementary file 2 — Table S1. Results of univariate analysis of progression free survival in patients with mCRC. (DOCX 16 kb) [file 12885_2018_5252_MOESM2_ESM.docx]

Supplementary Table 1- Results of univariate analysis of progression free survival in patients with mCRC

| Variables | Hazard ratio | 95%CI | P-value |
| --- | --- | --- | --- |
| Age (years) (≥ 65 versus < 65) | 0.721 | 0.488-1.064 | 0.100 |
| Gender | 1.212 | 0.904-1.627 | 0.199 |
| MPV (≥ 9.75 fL versus <9.75fL) | 1.170 | 0.860-1.593 | 0.318 |
| WBC | 1.041 | 0.980-1.107 | 0.190 |
| ANC | 1.057 | 0.989-1.130 | 0.100 |
| Lymphocyte | 0.925 | 0.707-1.210 | 0.570 |
| Hemoglobin (≤120 ×10^9^/L vs.＞120×10^9^/L) | 0.909 | 0.669-1.236 | 0.544 |
| PLT | 1.001 | 0.999-1.002 | 0.475 |
| PCT | 0.858 | 0.219-3.361 | 0.826 |
| PDW | 0.965 | 0.915-1.018 | 0.195 |
| PLR | 1.001 | 0.999-1.002 | 0.250 |
| NLR | 1.047 | 0.990-1.107 | 0.106 |
| Efficacy | 1.545 | 1.254-1.905 | 0.000 |

Abbreviation: see Table1 and Table 2.
